# A Window Into the Bright Side of Psychology: Interview With Mihaly Csikszentmihalyi

**DOI:** 10.5964/ejop.v13i4.1482

**Published:** 2017-11-30

**Authors:** Mihaly Csikszentmihalyi, Izabela Lebuda

**Affiliations:** a Claremont Graduate University, Claremont, CA, USA; b The University of Wroclaw, Wrocław, Poland

**Keywords:** Mihaly Csikszentmihalyi, positive psychology, interview

## Abstract

Mihaly Csikszentmihalyi is one of the most eminent psychologists of the modern era. His ideas, such as flow, or the systems model of creativity, have inspired numerous studies, theoretical analyses as well as pedagogic and psychological interventions. Alongside Martin Seligman, he founded positive psychology and continues to work to promote it. In this interview, he shares the stories behind his scientific interests, sources of scientific ideas and the process of promoting the concepts he had written about. He also shares his thoughts about academic work performance.

Mihaly Csikszentmihalyi is one of the most eminent psychologists of the modern era (Diener, Oishi, & Park, 2014). His ideas, such as flow, or the systems model of creativity, have inspired numerous studies, theoretical analyses as well as pedagogic and psychological interventions. Alongside Martin Seligman, he founded positive psychology and continues to work to promote it (Seligman & Csikszentmihalyi, 2000). In this interview, he shares the stories behind his scientific interests, sources of scientific ideas and the process of promoting the concepts he had written about. He also shares his thoughts about academic work performance.

**Izabela Lebuda:** *What is the achievement that you are proudest of?*

**Mihaly Csikszentmihalyi:** It depends on the point of view on my life. As a professional, I am proud of having helped psychology move into the direction of humanistic approach, into positive directions, which have been lost in the past. So that means the work on creativity, the work on flow. When I think about my achievements as a human being, not as a professional, I am the proudest of being able to have a life that I feel good about, with the children and my wife being so important. That is more important than the other kinds of achievements.

**Izabela Lebuda:** *I would like to ask you about your childhood. What kind of child were you? Did you like school? What were your interests and what did you like to do? Were you a well-behaved kid or rather not?*

[Contrib contnr1]**Mihaly Csikszentmihalyi:** Well… I don’t know. I remember that what I liked the most was probably lying in bed before I fell asleep or after I woke up in the morning and imagining things, day dreaming about being a warrior in the middle age. I remember that was a way to escape reality because home life wasn’t too good. My father was very busy, my mother, she was a very nice lady, but she didn’t know how to deal with children, so we had usually a German nanny. If the nannies didn’t work out, my parents used to let them go after 6 months, so the new nannies were coming and leaving all the time. I usually had more interaction with those nannies than with my parents. Nannies were not very imaginative; they just wanted you to follow the rules. Therefore, these stories that I imagined were partly based on my readings. I liked to read, I started when I was 6 years old. It was usually novels that little boys liked, Sir Walter Scott or Sienkiewicz, that kind of things. I didn’t go out to play with other children because we lived in an apartment in the middle of the city and there was nowhere to go. Occasionally we went back to Hungary for holidays. There, my grandparents had a place in the countryside near the mountains and Slovakia. That time was different but then I felt… In Italy, where we lived, they considered me a Hungarian, still a foreigner; they didn’t know how to understand me. In Hungary, they thought that I was an Italian. When the war came, we didn’t suffer much, because in Italy my father had a diplomatic status, which meant that each member of the family had three times as many tickets to buy things while everything was rationed. So we had enough food, we weren’t hungry, but life became even more restricted in a way. I was reading more, in fact my father thought I was reading too much, so he often said ‘No more books, no more books!’. Books back then were like television, like video games now. After the war, luckily I joined the Boy Scouts in Italy and that was very liberating. We went hiking, camping, mountain climbing, etc. Then I became a squad leader and troop leader so I had to learn how to deal with other people and motivate them to do things. I had to take a responsibility for them. From an educational point of view, that was more important than anything else. By the time I was 14 I left school, I didn’t like school anymore so I started working. I worked as a translator, then in a hotel in Milan. For some years I was a correspondent in Rome for the French newspaper *Le Monde*. I used to send articles and photography to them. So I did a little bit of everything, waiting in the restaurant my father built in Rome after he left the embassy. He was an ambassador until 1948 and then, after that, we had no place to go, we couldn’t go back to Hungary. In Italy there wasn’t much for him to do, he was already in his 50s, so he started a restaurant and I helped him there as a waiter. It became a very fashionable restaurant in Rome so we had all the American actors who came to do movies in Rome. It was like Hollywood, except cheaper, so a lot of American movies, with all the fights of gladiators, were done in Rome. I waited a table for Humphrey Bogart. And also the Hungarian football team that came to Italy to play was there. The Shah of Persia with his wife were there too, they were very nice. That was kind of fun and it was more interesting than school, so I left school and I did all those things.

[Contrib contnr2]**Izabela Lebuda:** *I’d like to ask you about leaving the school. Why didn’t you like it? What was the reason you left school?*

**Mihaly Csikszentmihalyi:** Well, you know, I think it was partly because schools were strange. After World War II, by the age of 10, you started Latin and the classes of Latin took 2 hours every day, from Monday to Saturday. Then, by the time you were 12 you stared Greek, another 2 hours every day. Ancient Latin, ancient Greek. Now I like Latin, I like to read in Latin, but at that time I thought it was a waste of time, it had nothing to do with the world, with what was happening around me. The teachers were very serious about the importance of studying Latin and it didn’t matter to me really. I saw during the war that many of my family members, the friends of family, who were well educated, were professors etc., how they were completely disintegrated when they lost their job, their title or money, so I thought: what good does it do to be so well educated and so knowledgeable about abstract things, how is it that when things turn bad you can’t rely on it? And you can’t say ‘Ok, I know what to do, doesn’t matter what happens outside, it matters how I experience my life.’ That was a kind of constant realization that made me think that schools were really missing more important things in life, they just got you to remember what happened in the past but didn’t give you the strength and the knowledge to face the future. So I started reading a lot, trying to find out myself what I could find that would be useful: religion, philosophy, literature, and so on. And there were good things in all of those disciplines but, on the other hand, they didn’t have a rigorous way of thinking about the issue, so you never knew if this is really true. Some things sounded true and were right, but I also saw the differences. On the one hand, science is building nuclear bombs and airplanes and rockets… And how much you can accomplish by disciplined thinking. I wanted to do something like that and try to understand human behavior so I was looking for something more modern, in a sense of more disciplined, more logical, and that was based on some knowledge of human behavior. Then, just by chance, when I was about 15, I saved some money so I was going to go skiing in Switzerland. I had enough money to travel there, in Zurich I stayed in a youth hostel (very cheap), then I was going to go to the Alps but it turned out that, that winter, spring came very early, so the snow was very mushy. It was better if you went up very high, but I didn’t have enough money to cover the expenses to go too far up. I stayed in Zurich and I wondered what to do, there were no good movies and I didn’t have money to go to the movies anyway. I saw in a Zurich newspaper a small advertisement that there was a free presentation, a talk by some Swiss professor on flying saucers. I thought that was OK, it was free, and it sounds like an interesting thing. I went there and the speaker turned out to be very different from what I thought. I thought he would talk about science fiction kinds of things but instead he talked about the need for Europe to find something to believe in after all the values had collapsed because of the war and that we have this real need to feel there’s something that makes sense. And we see the flying saucers because it is an archetype for unified knowledge and the values that Hindi call ‘The Mandala’. This sounded very strange but this professor was so precise and so logical in his exposition that I started buying and reading his books.

**Izabela Lebuda:** *Do you remember his name?*

**Mihaly Csikszentmihalyi:** Yes, it was Carl Gustav Jung. World famous, a star, student of Freud’s. So I started reading Jung’s books and they were not exactly what I was looking for but they had some elements of what was lacking in philosophy. Namely, observation of human behavior and trying to understand it and understand why these things happen, and so forth. After that I decided that maybe I should start studying psychology but, at that time in Europe, there was no psychology. You could take a course in psychology if you wanted to be a doctor or a philosopher. So I decided to come here (to USA) to study psychology. It took me about a year to get a visa. Finally, I got one and when I arrived in Chicago I had only $1.25 with me. There, the Catholic Relief Service found a place for me to stay with an old Hungarian lady, who was renting rooms to immigrants from Hungary. She gave me credit for first two weeks and I was supposed to find a job in that period and start paying. I found a job which I thought paid really well, but it started from 11pm until 9am. I wanted to go to school too so, finally, after 6 months I took the exams that gave me the chance to go to college. I hadn’t been in school for about 8 years but I passed because I had been reading so much. So I started going to school but I also worked from 11pm to 9am, so I would sleep only few hours and then go to school. That went on for 4-5 years, I had no life except work, sleep and school. I did my homework and the reading on weekends. It was interesting, challenging, although that was a bad time, I was very disappointed with psychology, because here, psychology was essentially about rats, everyone thought that Carl Jung was crazy. And that was my idea of interesting work, so it took time, many, many years so I could do the psychology that I was hoping to do. Now it’s much easier.  

**Izabela Lebuda:** *How did it happen that you managed to work in the area of positive psychology? What was the beginning of that? I believe that it was a hard time for positive psychology, nobody had interest in that at the time.*

**Mihaly Csikszentmihalyi:** Luckily, I came with that interest, that was my interest before I even knew that there was psychology. I knew that somehow we have to find a better life after the war. I was lucky in the war, I had a really good half-brother who was 10 years older and one who was 20 years older. The one who was 10 years older was killed and we were very close, he was killed during the seizure of Budapest. He was an engineering student at the university. They had 1400 engineering students at the University of Budapest and, as the Russians were coming in, they gave a gun to each of those students and told them ‘Now you are to protect this part of the perimeter’. After three weeks of fights there were only three of those students left alive, my brother wasn’t one of them. The oldest brother already had two children but he was captured by the Russians and taken to Siberia for six years. This just didn’t make sense, I don’t think anyone was happy with this way, with all the killing. People had to start again with nothing. It was such a wasteful and horrible way of living; on the other hand, humans could do all the other wonderful things. So that was the basis for my attitude towards knowledge, towards learning, it was to learn somehow to make life better. Psychology was trying to imitate physics and be very objective, but we can’t take humans as molecules and systems. It is a different problem with humans and trying to be abstract and scientific is not appropriate when it comes to understanding them. So I was concerned, all along I wanted to understand, to make a difference. Luckily, I started in a very simple and modest way to talk about play and playfulness, feeling the freedom and enjoyment that comes in ways that we can feel. Why can’t we have a life like before? That was the simple question that hasn’t been asked by psychologists. For instance, originally, my first understanding of ‘flow’ came from teaching a class in a college I was teaching at in 1968-69. While teaching a class, I asked my students ‘what should we focus on this term?’ and I gave them several choices, one of which was ‘play’. And they said ok. This was in the spring and the course was to start in September, so between spring and the fall I read everything I could find on play in psychology. It was strange because all the great psychologists like Erikson, Piaget and so forth, they wrote about play, but they wrote form a very strange perspective. For me it was strange, because they always looked at play as a practice for adult life, chess makes you think as an engineer or accountant, basketball makes you healthy and makes you learn group cooperation. You learn social graces by pretending to be a doctor. So it is all utilitarian and it develops competences as an adult. And it didn’t make any sense to me, because I think children play to feel in control, they develop skills, they master something and they feel good about that. It is a different perspective, so we tried to describe it. I asked my students to study some play but with adults, not with children, so they found very strange people who spend time doing very strange things. Mainly it was sports, music and things like chess or bridge, poker or something like that. One thing I told them to ask was ‘how does it feel when they are doing it?’. Just describe it phenomenologically, how it is experienced as you do this. After three weeks or so, they came back with interviews and we started analyzing the themes from the interviews. It was very strange, as I didn’t expect this, but whether you talk to a hockey player or a musician or chess player, whatever they said they loved to do, they described it in a similar way. They talked about the things like: ‘I like to do this because I am good at it, I meet challenges, I find this thing challenging and I feel good when, through my skill, I am able to maser the challenge. I know clearly what I have to do, I get constantly the feeling of knowing how well I am doing’. Then I realized that is it not so much the form of the play that is important, but it is the experience of playfulness that people have when they do it. It took 2-3 years before I gave it the name ‘flow’ because ‘flow’ was very often mentioned by people (‘Oh, I am being carried by the river, I don’t have to think, I just do it, spontaneously, automatically’). ‘Flow’ was the name that came out, as I say, it was a very simple thing, it was something that I did for a class but it resonated so much with people, after a while. It became something that people talk of as if it was a really important thing, which I think it is, but I am surprised that it came out like that.

**Izabela Lebuda:** *What was your first experience in the scientific arena with this new area of study in psychology? What was the reaction of the other psychologists? How did they react to your area of interest with their “serious” approach to psychology?*

**Mihaly Csikszentmihalyi:** It took probably almost ten years for psychologists to notice it. The first ones to notice it were the anthropologists. A couple of anthropologists who were famous said ‘Yes, that is what we noticed when people danced around the fire in primitive societies’. They wrote it down and they started to use this term. Then, the second group was sports psychologists, they noticed it in the subjects they studied. These were the first two groups that started writing about flow. And then there was not much interest, a few psychologists would write about it but without too much repercussion in psychology. That was between 1975 and 1990, so for 15 years it wasn’t really accepted or noticed. But then, for some reason, a journalist in New York read some of my work on ‘flow’, which was not read by many people, and he published an article in New York Times Health Section. That was nice, I was very pleased, it was interesting. Then I got a letter from a literary agent from New York, who read the article and decided it was a very interesting issue and asked me to write a book about it. I told him I didn’t have enough data for the book. He said he didn’t mean ‘that’ kind of book, he wanted me to write a book for normal people, intelligent people, who even without the data would recognize the idea because of their own experience. At the beginning I didn’t take it seriously but then after a month or so, he sent me a list of all the scientist for whom he was an agent, these included some of the great physicists, biologists, chemists, astronomers, etc. It looked interesting, I thought that maybe I should try it. So, I signed a contract with him and I began to write a book on ‘flow’. As a “standard” writer I felt liberated that I didn’t have to do the micro analytic work that you have to do to publish in journals. It was a great experience, I enjoyed writing but I didn’t think that much would come out of it. Before I started writing the book, the agent from New York asked me to send two pages of description of what I would write when I was ready. After a period of thinking I wrote it for him. After two weeks, he started auctioning the proposal to publishers. I didn’t think I knew what that meant, but he explained he would take it to ten of publishers in New York and say that they have two weeks to make an offer. If one of them said that he would buy it for $10000 the agent would call up the other publishers up to inform them about the offer so they would come up with a higher one. He started doing that and, in the meantime, with my wife Isabella and our two kids I went to Europe, visited friends in Switzerland and so forth. Every afternoon I would go out with a lot of metal coins to put in a public phone. Then I would call the agent and ask ‘What is happening?’. And he always replied ‘It is all going well’. About five days later he said that we were ready, HarperCollins offered $70000, we couldn’t get more than that. So now I had to write the book. When I started writing it was really fun and I am very glad now that it was published. That agent is a very interesting man actually, John Brockman, he publishes many things, collections of authors’ work. Every year, in the middle of December, he asks the people whom he represents a question like ‘What are you optimistic about?’. Some of these people are well known, Jerry Diamond, he is a very important writer, Stephen Pinker, famous psychologist. There are all these various essays, they are all very short, they are supposed to be very short. I have two or three of these books. One is on childhood ‘How did you become a scientist?’ He is interesting in terms of how to spread ideas quickly. He has all these antennas open to the most interesting things in several fields, mostly science. And then he can help transfer those ideas from the domain into the culture. So he is a connector in that way. He is unique, I have to say.

**Izabela Lebuda:** *I would like to ask you about people: who helped you develop your scientific interests, scientific creativity?*

**Mihaly Csikszentmihalyi:** I was lucky that, at the University of Chicago, I had a professor who came from Poland. He was from a Jewish family, his name is Jacob W. Getzels. He had just published in 1956 a book called ‘Creativity and Intelligence’. It was the first study that showed that these two things are pretty orthogonal. You can be high on both, or low on one, high on the other, or vice versa. That was a very influential book in education. Especially because it showed that children who are high on intelligence but low on creativity are considered to be much better that children who are high on creativity but low on intelligence. When I saw how little there was to do in psychology that was interesting I thought that this area is interesting for me. So we worked together, he was supportive and he was a good example of a real scholar. There’s an English expression ‘a scholar and a gentleman’ and he had those qualities. When he died, about 10 years ago, his wife asked if I could write an obituary for him in the New York Times. It was good to be able to do that, he had been a really good mentor.

Even though the first class I took with him was a class on values, the psychology of value. On the first day, there were maybe 15, 16 students, graduate students, all sitting at the table. He asked each one of them to explain why they took that class, why they signed up for that course. And when he came to me, I said ‘I am not sure if value still matters much or if there is anything interesting about it. But I had had free time in that period and I thought I would take it’. When I said that, he just pointed at the door and told me to leave the room. So, for a while I avoided him. But after a while, he knew that I did some good work in other classes. He was quite brusque too, he would ask me ‘Why don’t you come and talk to me?’ Once in an elevator I actually told him that I would like to do some work on creativity. I think he didn’t answer then, but two weeks later said something. After that, we really worked well because I respected his integrity, intellectual integrity. I was a fast worker, I could do things very quickly. It is because I worked for a newspaper for so many years. When something happened in Rome, I had to write it down and quickly send it to Paris. That ability to focus and try to get to the central issue and work quickly – that is something I learned from journalism. It helped me in graduate school a great deal.

So that was our collaboration (with Jacob W. Getzels): we wrote one book together and many, many articles, about 15 or so. When I left, when I got my PhD I got several offers, including from Harvard. But Harvard had a bad reputation because they hired smart, young people, kept them for 3-4 years, then they let them sign off and get new ones. Harvard wasn’t interesting, some of the others weren’t either. But, instead, a friend of mine was teaching in a college in the Department of Sociology and Anthropology, so he asked me to come to see him and talk. So I drove outside Chicago, two hours away, not that far but it is in a beautiful place, right by the lake, with beautiful lawns and huge houses. It was a small college (Lake Forest College) but a very nice one, where the students were doing very interesting things. You know, it was around 1965 when I finished, with all the student movements happening. And there they had the Department of Sociology and Anthropology which was really on the edge of this new, changing spirit of America and Europe too at the time. They offered me a job there in Sociology and Anthropology, a topic I had never studied but I took that job. It was fun because every year I learned more and more about these topics. That was a period of about six years there, it was really good because I was free there, I could do things that were not sociology or even psychology, it was more creativity that I was doing there. That is why, at the beginning, as I said, it was mostly anthropologists who were interested in my idea. I was thinking like a sociologist or anthropologist for a while.

My background in journalism was very helpful. Then I met some people whom I didn’t know that well but were very helpful, for instance the psychologist Donald T. Campbell. He was the first psychologist who paid attention to me. Donald T. Campbell became the president of the American Psychological Association. He was very well-known; we became friends, he really helped me by mentioning my work and saying how important it was. He was the first psychologist to do that.

**Izabela Lebuda:** *What did your parents think about your decision of leaving school and going abroad? What was their reaction to your choice of a not very popular type of science at the time? Did they have anything against your life choices?*

**Mihaly Csikszentmihalyi:** My parents had been kind of shocked by the war. They had lived a comfortable and good life but after the war they were not sure what was the right thing to do. When I decided to leave school they couldn’t tell me what was better to do. I moved away from the family. One of my jobs when I was just 15 was to work as a translator in one of the Institutes of the University of Rome. They had a big, beautiful building in a park. Because of one of my friends from Boys Scouts, whose father was a head of this Institute, they offered me to stay there for free, I could eat there and sleep there. I was sleeping in a huge bathroom that wasn’t used in one of the corridors of the hospital. They put some wood planks and the mattress on the bathtub and that was my bed. The only problem was that, occasionally, there would be a rat coming out. Once I woke up and a rat was biting my nose. They gave me breakfast and lunch. I translated medical stuff for them, I hope nobody got hurt because I didn’t know if I was doing it right. I was trying to improvise a little bit. They didn’t know much English at the time, very few people in Italy knew English. I didn’t know much either but I knew German, and I figured that it is similar to English. Anyway, that left me free, I did the translations but otherwise I was free, so I could do other jobs, do a lot of reading. I was pretty happy so my parents were ok with that. Then, when I left for the US, my parents were glad that I planned to start studying again. But then they moved to Belgium. You asked me what did my parents think. Well, at the age of 14 I was no longer living with them. My parents stayed in a kind of refugee camp, I was there for some time too but then I left to work. They stayed in this camp until they went to Belgium. They couldn’t really help me much, so I had to find what I could do, given that there was not much I could do.

**Izabela Lebuda:** *You have mentioned Isabella, your wife, and your sons. What do they think of your work? Do you work with them?*

**Mihaly Csikszentmihalyi:** Yes, occasionally we get invited to speak together. My oldest son, Mark, who is Chair of the Department of Far Eastern Civilizations at Berkley, and I had a conversation with all the psychologists at the Second World Conference on Positive Psychology about solitude and its consequences in China. I appeared with my youngest son at two or three conferences where he was talking about his robots (he is a Professor of robotics at the University of Madeira) and I was talking about flow and creativity. They don’t want to do it too often because they don’t want to consider that they are kind of an opening act for my appearance. And they are also a little suspicious of psychology, although both of them are good scientists. So we have quite long arguments about evolution and so on. I respect both of them, they are fantastic, serious, committed, very uninterested in materialistic things, even less than we are. They have their practice, they are not religious or spiritual, and that’s because of their secular humanism. They are interesting people.

**Izabela Lebuda:** *What was the most important thing to teach them while they still were small children? And now what do you want to pass on to your grandchildren?*

**Mihaly Csikszentmihalyi:** I think one of the things is not to be afraid of the world and to do what you think is important. Do not let people push you around and make you think differently from what you believe. Be independent and yet love other people, I mean, be open to other people. So this kind of balance. In some things it is important to be balanced but in the others you just think on one side, you can’t go back and forth to certain issues. But most of life is not like that, throughout most of your life you have to realize that you don’t know enough, that you are prejudiced, that you have a wrong understanding of things because of your background, ethnic group, whatever. Then you should be able to listen and empathize with others.

**Izabela Lebuda:** *As you have mentioned balance, I would like to ask you: how important for you is the balance between your work and your private life? Is it important, helpful? Or would you rather agree with some of eminent people that you have to make a choice between these two things?*

**Mihaly Csikszentmihalyi:** I try to balance as much as possible. I married Isabella in 1961 and I think that for the first 20 years of marriage I had to work very hard outside. She didn’t like Chicago once we moved back there. She thought it was dangerous and confusing and cold. Then, when we came once here (to California) for a year, she loved it so much, the winter here, that we decided she spent already too much time in Chicago so I would move from Chicago. I didn’t want to move, it was not a good choice for professional reasons to come here. But I figured she was happy here and I could be happy too. It wasn’t my choice but I was ok. I don’t regret it, I am glad that we moved. It meant a whole different type of work, trying to make this positive psychology work out which is not really my strength. I am not an organizer or a planner. But I think somebody had to do it. Now I am still away most of the time but at least Isabella is much happier now when I am not here because it is a nicer environment.

**Izabela Lebuda:** *How does you usual day look like? Are your days usually similar, do you have your daily routine?*

**Mihaly Csikszentmihalyi:** Of course I try not to have routine days but then I do. Somehow each week there are two classes that I teach, they structure the rest of the week because I have to prepare for it two days in advance. After that I have to read the papers that students write. So those are the routine parts. Around it varies a lot. One thing about this school here, which is different from Chicago, is that there are very strong expectations for students. It’s up to the professor that they make sure they do it. That is so different from Chicago, it’s incredible. This is better for people who are not scholars, who are interested in doing things. In Chicago they just wanted to stay students, the average time from entering the graduate school and finishing it was 16 years. That is just because students just love to be students. They would go to Tibet or South America for a year, come back. They read, they write, they also teach. They are like medieval scholars. It is nice if you can afford it.

**Izabela Lebuda:** *Do you like teaching?*

**Mihaly Csikszentmihalyi:** I like to teach students who are really interested, that’s the problem. I started teaching business students, even when I was in Chicago they asked me to give some courses. And there the students were mostly older people, who were already working. I would come in, start talking about interesting things conceptually and within 5-10 minutes almost all of them were reading the Wall Street Journal and taking notes from it. I realized that they need something else, they don’t know what to do with what I say. So I tried to change my teaching there and it became more interesting for them. They began to appreciate it and that was good. When I came here (to California) originally I was hired by the Drucker School of Management, which is all about business and I liked that, I liked their students. I like business people, I like to teach them, but none of them wants to do research so that was not so good for me and I didn’t enjoy that part. I felt like I was just repeating things and stagnating. So, then I decided to offer the Psychology Department this new positive psychology idea, and they were interested.

**Izabela Lebuda:** *What would you advise young people who want to develop in science? What should they do, how should they plan their career?*

**Mihaly Csikszentmihalyi:** I have a slide (in teaching) with a famous composer of music telling his students: ‘Don’t do it for the money, don’t do it to be famous, do it because you love to do it, otherwise is not worth it’. I believe that is 100% true. In music that is the only thing but in science it is not the only thing. You can really make a good career, make money and change things. But still, there are many other things to motivate you, like money and an interesting new job. Of course, ethics is important, which you often don’t learn until it’s too late. I am surprised by how many of the scientists, Nobel Prize winners who I interviewed, had comfortable, satisfying lives. At some point of their career maybe they were very aggressive but basically they just enjoyed understanding how the world works. And trying to add something to the knowledge-base of humanity. But otherwise, they had kind of a bourgeoisie existence. So, it is a good career if you love it, it can’t be better.

**Izabela Lebuda:** *What do you think is crucial in science? Intellectual, personal or social skills?*

**Mihaly Csikszentmihalyi:** Ideally, they are all important. The social one is important to get ideas from many people, talking to them just informally and getting ideas. Also it is important to get other people to listen to you and your ideas. If you are sociable you can present your ideas in more interesting and convincing ways. As for the personal skills, what you need is curiosity and perseverance. If you don’t have those two traits you are not going to go very far. Those things are a little bit conflicting, although not that much. Intellectual skills are less important than you would expect but still quite vital. I think that personality and social skills are almost as important.

**Izabela Lebuda:** *I would like to ask you a question that is very interesting for me. How do you manage to write so many publications? There’s so many of them and each of them is highly fascinating and helps me discover something completely new.*

**Mihaly Csikszentmihalyi:** Well, I think partly this was also the heritage of journalism. I always liked to write, I liked to write even at school when we had essays to write. And those were things that my teachers liked best when I was doing it. I think that nobody in my family… My mother wrote, she wrote a history of the world from a religious point of view, the evolution of religion and so on. However, she wasn’t educated, I mean she graduated from high school but never went to college. She read a lot, and wrote a lot, I believe I must have inherited some of that because I like to write.

There are many people who wrote more than me, in psychology for instance Sternberg or Simonton. But they are much more specialized, I mean they only write on the same topic. What I like about the books and the articles I write is that each of them is about something different, that is very enjoyable. I don’t get bored, it is a challenge for me.

**Izabela Lebuda:** *And do you think that scientists should have very broad interests or rather very focused, specialized ones?*

**Mihaly Csikszentmihalyi:** The advice that people usually get when they go to graduate school is that you should write a lot about small things. You have to become known for being the best at a particular topic. And then, after probably 10 years you can write about anything you want. However, I started to write about the things I wanted to write about from the beginning. My first publication is about how in different cultures you have different ways of drinking. It was called ‘Socio-cultural differences in group drinking’. It was interesting, because you can find so many different stories. When Hitler started building the national-socialist party, he started in some wine shops around Munich, where the people used to go, in the open air with grapes and stuff. There were all these little tables and families sat around them, ate and drunk. He would stand there somewhere on a podium and try to talk to them. He found out that it didn’t work, people listened but didn’t really pay attention. So after a couple of years he started to hold his meetings in the beerhouse, which were kind of underground, low ceilings, long tables and people sat at these tables. They were too far from each other to talk and he was at the podium at the end, when he started talking, people turned and they had nothing else to do but listen to him. That change the whole chemistry, the dynamics of the presentation, and that was interesting.

Then you look like at France the impressionist painters in France who used to meet at little coffee tables and talk to each other all the time. The interesting thing was also how important it is in history, not just in the West, when people eat a lot and drink a lot. There were times when the social structure was about to change, for example when a new archbishop in England was appointed, he invited everybody and they would eat and drink all week. The same thing was when a King married a Queen from another country, where there was not much trust between people, they had a big party and everybody was happy afterwards. For instance, when the Dutch became very important and rich traders, in the 1600-1700, they found that doing much of the business with other merchants at tables when they ate and drunk, that smoothed things out. It was so successful that the mayor of Amsterdam had to put a new law that in effect you couldn’t hold a lunch for that lasted more than four days, or a dinner more than seven days. Because the people just slept under the table. But the church got really upset that people were just drinking and eating all the time so they put pressure on the mayor to change the law.

Then, for instance, how the bars, the American bar came up. They began to appear in England and then in America during the industrial revolution, when suddenly all the people came before the opening of the factory and then left together after closing, and they had to serve hundreds of people and take it to the tables. So, instead of tables, the bar became an assembly line for drinking.

**Izabela Lebuda:** *I believe that this interest motivates you to work, but maybe there is something else that also stimulates you to work?*

**Mihaly Csikszentmihalyi:** It is a good life but it is very hard actually. It is something that you can continue doing after you are very tired, you don’t have to stop thinking, you can still write. That gives you the skills to do things that are important. I worked so much before, when I went back to school. I worked for many years, so I knew what work was like from the inside. I knew that the kind of work that I could do was teaching and doing research at university. It was better than anything else I could think of. Well, actually I had a question just before finishing by PhD, before I passed my final exams. At that point I felt that it was too much, too much hassle, too much pressure. I was thinking that maybe I should have chosen some regular job. Because I published two stories in the New Yorker, which is considered to be the best magazine in USA. I wrote to them asking whether they had some job as editor because I would like to do it. I like to write, I like to think of how to improve the writing of others and so forth. Luckily, the New Yorker wasn’t looking for anyone at that time.

**Izabela Lebuda:** *Do you feel that you influence other people’s work?*

**Mihaly Csikszentmihalyi:** Yes, it is a nice feeling. Last week there was an article in the *New York Times* that I am getting so many emails about. It is an article by a composer who describes how ‘flow’ helped him to become a composer.

**Izabela Lebuda:** *Do you feel successful?*

**Mihaly Csikszentmihalyi:** Frankly, I don’t think much about that. It was much easier to think about ‘flow’ before I wrote the book, the most popular of my books. Now, to get any kind of free time is really hard. Before I could do whatever I wanted to do, now I have too many things to do. But on the other hand, I figure that, hopefully, some of it will be of use to others, that I will have time to travel with Isabella for a year before it is too late. We have been traveling a lot, still, for instance recently we spent five weeks in Northern Italy, just the two of us, and our kids came as well as several grandchildren. I had my computer with me but I didn’t really use it. Otherwise, it was all free time.
